# The effect of homelessness on viral suppression in an underserved metropolitan area of middle Tennessee: potential implications for ending the HIV epidemic

**DOI:** 10.1186/s12879-022-07105-y

**Published:** 2022-02-10

**Authors:** Vladimir Berthaud, Livette Johnson, Ronda Jennings, Maxine Chandler-Auguste, Abosede Osijo, Marie T. Baldwin, Patricia Matthews-Juarez, Paul Juarez, Derek Wilus, Mohammad Tabatabai

**Affiliations:** 1 Division of Infectious Diseases, Meharry Community Wellness Center, Meharry Medical College, Nashville, TN USA; 2 School of Graduate Studies and Research, Meharry Medical College, Nashville, TN USA; 3 Department of Family and Community Medicine, Meharry Medical College, Nashville, TN USA

**Keywords:** Homelessness, HIV, Social determinants, And viral suppression

## Abstract

**Background:**

A wealth of scientific evidence supports the effectiveness of HIV prophylaxis and treatment. Homelessness is strongly associated with the health status and viral suppression among underserved populations and can undermine the national plan to eliminate HIV by 2030. This retrospective observational study examined the extent in which homelessness affects HIV treatment in an underserved urban area of Middle Tennessee in 2014–2019.

**Results:**

Among 692 HIV-seropositive patients, the proportion of homeless patients increased from 13.5% in 2014 to 27.7% in 2019, thrice the national average for HIV-seropositive people (8.4%) and twice that of HIV positive patients who are participating in Ryan White programs nationwide (12.9%). Our findings suggest that homeless patients were half as likely to achieve viral suppression as compared to those who had a permanent/stable home [OR 0.48 (0.32–0.72), p-value < 0.001].

**Conclusion:**

Our study indicates that homelessness may play an important role in viral suppression among persons living with HIV/AIDS in Middle Tennessee.

## Background

HIV has become a chronic condition, easily manageable by taking one multi-drug, single daily pill. However, the HIV epidemic continues to affect predominantly minorities of color, underserved and marginalized populations including persons experiencing homelessness. The Center for Disease Control and Prevention (CDC) estimates that 1.2 million people aged 13 and older were living with HIV in the United States and 37,832 persons were newly diagnosed HIV-seropositive in 2018 [1]. Of them, Black/African American represented 42%, while they account for just 13% of the U.S. population. Overall, the number of new infections remains unacceptably high, especially among men having sex with men (MSM) in the age group 25–34, while there has been a 42% increase in the incidence rate between the Black/African American and Hispanic/Latinx racial groups.

### Homelessness and HIV-related health outcomes

HIV-seropositive persons are disproportionately overrepresented among the homeless population. In 2016, the CDC reported that 8.4% of people in HIV medical care were homeless [2]. A qualitative review of 17 published papers examining the effect of homelessness on health status, HIV treatment adherence, and health outcomes showed that homelessness is highly prevalent among Persons living with HIV/AIDS (PLWHA) and strongly associated with poorer health status, lower adherence to antiretroviral therapy, and worse CD4 cell count and viral load outcomes [3].

### Homelessness in Nashville, Tennessee

During the past 5 years, in Nashville, Tennessee, the trend of homelessness paralleled the economic growth curve that propelled a booming housing market and aggressive gentrification. In addition, the trauma, poverty, and stigmatization will exasperate the homelessness epidemic in the US and access to appropriate healthcare. Nashville officials located approximately 2300 people experiencing homelessness on an overnight count in 2017. However, local advocates estimate the number of people in Nashville living on the streets, in cars, camps, motels or in shelters at 20,000 [4].

The purpose of this study was to evaluate the effect of people experiencing homelessness on viral suppression in persons with HIV/AIDS or PLWHA in an underserved area of Middle Tennessee in 2014–2019. We hypothesize that people experiencing homelessness will have worse virologic outcomes compared to those individuals living in stable housing.

## Methods

### Study setting

Meharry Medical College is located at the epicenter of the HIV epidemic in North Nashville, Davidson County, in Middle Tennessee. Thirteen counties including Davidson make the Nashville Transitional Grant Area (TGA) of the Ryan White Part A program. While about 39% of the TGA’s population lives in Davidson County, 76.5% of the PLWH population resides in Davidson County. Twenty percent of them had been in jail, 42% homeless/unstably housed at some point in the last year, while 40.2% did not have food to eat three or more days at some point in the last year, according to Nashville Metro Health Department statistics. As a Tennessee-designated AIDS Center of Excellence, Meharry Community Wellness Center (MCWC) has been a major provider of comprehensive, integrated, and patient-centered care and health services for the most underserved PLWH in Davidson County since 2005.

### Study design

We conducted a cross-sectional analysis of 692 HIV-seropositive adult and adolescent patients seen at MCWC between 2014 and 2019. The Meharry Institutional Review Board (IRB) had approved this study.

#### Inclusion criteria

All adult and adolescent patients who had at least one medical visit at MCWC between January 1, 2014 and December 31, 2019 were included.

#### Exclusion criteria

From this study analysis, we excluded prison inmates of Tennessee Department of Corrections, seen through our telemedicine program and jail inmates who had face-to-face clinic encounters.

#### Data sources

Data elements extracted from CAREWare electronic patients’ charts included the following: age group, race/ethnicity, gender, HIV risk factor, federal poverty level (FPL), type of medical insurance, housing status (permanently housed and homeless), rate of clinic visits per month, last recorded CD4 cell count and plasma HIV viral load. CAREWare is an electronic health and social support services information system for HRSA’s Ryan White HIV/AIDS Program recipients and providers. MCWC staff entered all data elements in CAREWare at each patient visit. We performed data quality check, for accuracy, duplication, missing, and unknown elements before generating the study database. No personal identifiers were included. Nearly all viral load and CD4 cell count results were imported from our contractual commercial lab web portal and very few of them came from external providers’ reports.

### Measures

#### Primary outcome variable

Viral load suppression, defined as plasma HIV viral load below the detection limit of 20 copies/mL measured by Real Time PCR assay, constituted the primary outcome variable. Of note, our viral suppression threshold is lower than commonly used 200 copies/mL in published reports. The outcome variable is coded as 0 for virally suppressed and 1 for not virally suppressed.

#### Independent variables

We chose a pool of independent variables from patient’s electronic medical records (CAREWare 6, version 58). Demographic variables consisted of age group (18–24, 25–44, 45–64, and 65 +), gender (male, female and transgender), and race/ethnicity (Black or African American, non-Hispanic white, and other including Hispanic/Latinx). Variables of social determinants of health comprise HIV risk category (heterosexual, MSM, and/or bisexual men, injection drug use or IDU, and other), housing status (homeless or stable), FPL, and type of medical insurance (no insurance, Medicaid/Medicare, private insurance and other). Homeless was defined as people living in shelters, transient homes, in the streets, or unable to pay rent or mortgage. We entered modified adjusted gross income (MAGI) as the percentage of FPL, computed automatically by CAREWare, and we analyzed it as a continuous variable. As a payor of last resort, the Ryan White Program provided ambulatory/outpatient medical insurance coverage to eligible, low-income, uninsured clients. We determined retention in care as the rate of monthly medical visits. The last recorded CD4 cell count and plasma viral load were collected for this study. We treated CD4 cell count and viral load as dichotomous variables (< 500 or ≥ 500 cells/mm^3^) and (≤ 20 or > 20 copies/mL). We considered plasma viral load ≤ 20 copies/mL as viral suppression.

### Statistical analysis plan

First, we performed exploratory data analysis and checked for data quality, distribution, and satisfaction of model assumptions. To build the model, we first entered the binary homelessness variable using a univariate binary logistic regression with its corresponding odds ratio. After that, we constructed a multivariable binary logistic regression and calculated the adjusted odds ratios with their 95% confidence intervals to find the effect of homelessness on viral load suppression. Then, in the multivariable model we controlled for demographic, social, and clinical variables: age group, gender, race/ethnicity, type of medical insurance, FPL, HIV risk factor, rate of outpatient and HRSA visits, and CD4 cell count. Finally, we plotted a receiver operating characteristic (ROC) curve, which summarizes the tradeoff between the true positive rate (sensitivity) and false positive rate (1-specificity) for our logistic model using different probability thresholds. The area under the curve (AUC), an aggregated metric that evaluates how well our logistic regression model classifies our binary outcome variable viral load suppression at all possible cut-offs, was computed. The AUC ranges from 0.5 to 1. The higher the AUC, the better the model is at distinguishing between patients that are virally suppressed and patients not virally suppressed. IBM SPSS version 27 was used to perform all analyses, including graphics.

## Results

### Population characteristics

Table 1 summarizes the main characteristics of study population. From 2014 to 2019, this study enrolled 692 HIV-seropositive patients, 498 males (72%), 184 females (26.6%), and 10 transgender (1.4%). The mean follow-up period was 5.42 (SD = 4.54, 95% CI: 5.08–5.76) years and the median follow-up time was 4.32 years. The majority of the patients (79.6%) were Black/African American, 15% were non-Hispanic White, and 5.3% belonged to another race category, almost all of them identified as Hispanic/Latinx. Youth (age 18–24 years) accounted for 5.8%, while the age groups 25–44, 45–64, and 65 + years represented 41.5%, 46.5%, and 6.2% of the study population respectively. Most of the patients (69.1% in 2014 and 60.5% in 2019) lived below 100% of the Federal Poverty Level (FPL). In relation to HIV risk factor, 46% of the patients reported as heterosexual in 2019, a sharp decrease from 62.4% in 2014. The proportion of patients identifying themselves as MSM increased from 34.8% in 2014 to 42.8% in 2019. This reflects the current epidemic trend in the South. The proportion of patients who considered IDU as their HIV risk category decreased from 11.4% in 2014 to 9.4% in 2019, reflecting the epidemic shift in opioid addiction, from inner cities to suburban and rural areas, and from Black/African American to White/Caucasian communities. This observation correlates with the overall decrease in the number of clients tested with chronic hepatitis C infection from 74% in 2014 to 57% in 2019.

**Table 1:** Frequency table of HIV-positive patients by housing status

	Total	Permanently housed	Homeless
Unweighted N	692	500	192
Gender			
Male	498	70.8%	75%
Female	184	27.6%	24%
Transgender	10	1.6%	1%
Race/ethnicity			
Black	551	79.2%	80.7%
White/non-Hispanic	104	14.4%	16.7%
Other	37		
Age group			
18–24	40	5.2%	7.3%
25–44	287	42%	41%
45–64	322	45%	50.5%
65 +	43	7.8%	2.1%
HIV risk factor			
Heterosexual	318	45.4%	47.4%
IDU	65	8.8%	10.9%
MSM	296	44.6%	38%
Other	13	1.2%	3.6%
Insurance type			
Medicaid	160	22%	26%
Medicare	93	15%	9.4%
Ryan White	260	32%	52.1%
Corrections	14	3.1%	
Private	165	28.4%	12%
CD4 cell count			
< 500	315	42.6%	53.1%
≥ 500	377	57.4%	46.9%
HIV viral load			
Suppressed	477	78%	22%
Not Suppressed	215	25.6%	45.3%

### Main/primary clinical outcomes

Testing for the essential biologic markers of antiretroviral treatment revealed that 54.5% of the patients had a robust CD4 cell count ≥ 500 cells/mm^3^ (HIV infection, stage 1) and 68.9% of them achieved plasma viral load suppression below the detection limit of 20 copies/mL. Permanently housed patients had a much higher proportion of viral suppression compared with their homeless counterparts. As shown in Table 2, the odds of those not virally suppressed for persons living in a stable house compared to persons in an unstable house is 0.42 with a 95% confidence interval estimate of (0.29, 0.59) and the adjusted odds ratio is 0.48 with a 95% confidence interval estimate of (0.32, 0.72). Among patients with viral load less than 20 copies/mL, 78% were permanently/stably housed and 22% were homeless, while 74% of permanently/housed patients reached viral suppression and a much smaller proportion of the homeless patients (54.7%) remained virally suppressed.

**Table 2:** Odds ratios and adjusted odds ratios with their corresponding 95% confidence intervals for binary viral load outcome

Variable name	Odds ratio and 95% CI for odds ratio	P-value	Adjusted odds ratio and 95% CI for adjusted odds ratio	P-Value
Housing status				
Unstable housing	1		1	
Stable housing	0.42 (0.29, 0.59)	< 0.001	0.48 (0.32, 0.72)	< 0.001
Gender				
Transgender	1		1	
Female	0.78 (0.19, 3.13)	0.724	0.68 (0.13, 3.66)	0.651
Male	1.17 (0.30, 4.57)	0.825	1.00 (0.20, 4.98)	1.000
Race/Ethnicity				
Other	1		1	
Black or African American	1.26 (0.60, 2.65)	0.549	1.52 (0.65, 3.56)	0.334
White (non-Hispanic)	1.10 (0.47, 2.54)	0.833	1.39 (0.54, 3.60)	0.498
Age group				
65 years old or older	1		1	
18–24-year-old	0.98 (0.37, 2.57)	0.967	0.83 (0.26, 2.70)	0.760
25–44-year-old	1.54 (0.76, 3.12)	0.235	1.70 (0.70, 4.17)	0.244
45–64-year-old	0.93 (0.46, 1.89)	0.833	0.77 (0.34, 1.76)	0.534
HIV risk factor				
Other	1		1	
Heterosexual	1.44 (0.39, 5.35)	0.585	2.63 (0.58, 11.98)	0.212
IDU	1.48 (0.37, 5.97)	0.580	2.43 (0.49, 12.07)	0.277
MSM	1.60 (0.43, 5.95)	0.483	2.35 (0.53, 10.50)	0.264
Insurance type				
Private	1		1	
Medicaid	1.67 (1.02, 2.73)	0.043	1.16 (0.62, 2.18)	0.638
Medicare	1.07 (0.59, 1.96)	0.821	0.83 (0.40, 1.74)	0.623
No insurance	2.13 (1.37, 3.31)	< 0.001	1.21 (0.70, 2.09)	0.486
Corrections	1.92 (0.61, 6.09)	0.267	0.90 (0.24, 3.41)	0.881
CD4 cell count				
CD4 ≥ 500	1		1	
CD4 < 500	4.65 (3.28, 6.59)	< 0.001	4.93 (3.38, 7.21)	< 0.001
Poverty level	1.00 (1.00, 1.00)	< 0.001	1.00 (1.00, 1.00)	0.031
Rate of HRSA visits	1.01 (1.00, 1.01)	< 0.001	1.19 (0.99, 1.42)	0.059
Rate of outpatient visits	1.01 (1.00, 1.01)	< 0.001	0.84 (0.70, 1.01)	0.066

**Fig. 1:**
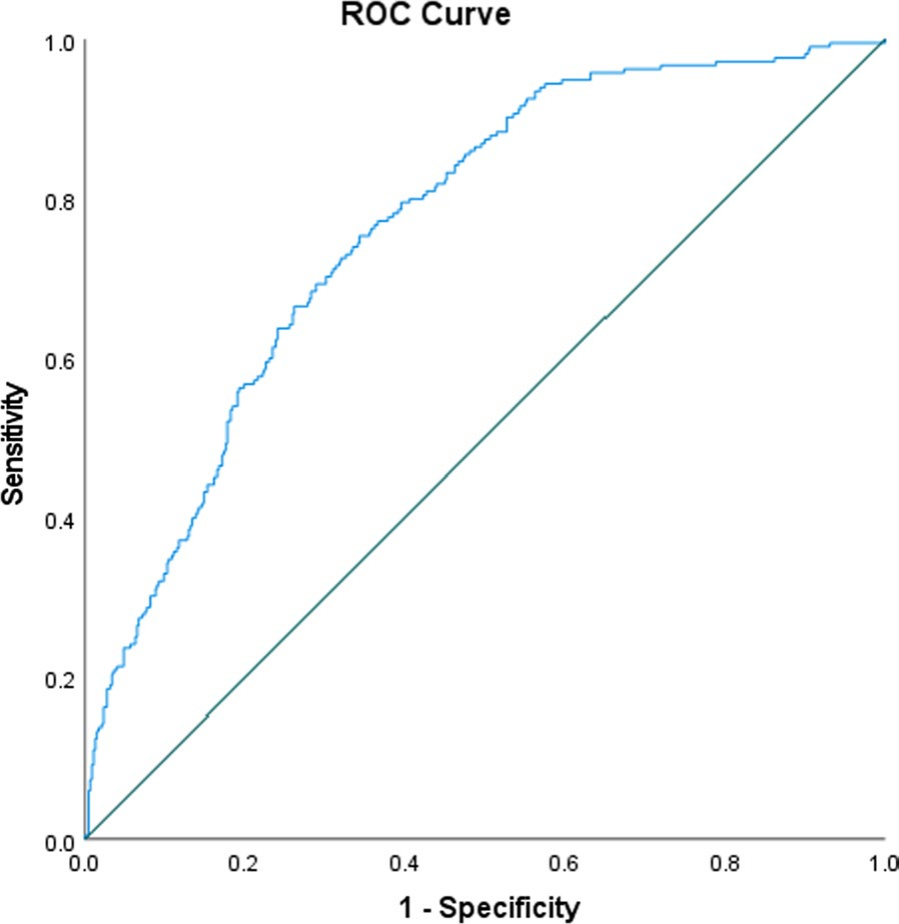
Receiving Operating Characteristic (ROC) Curve

The multivariable binary logistic regression demonstrated that permanent/stable housing (p-value < 0.001) was a significant factor of plasma HIV viral load suppression. It also revealed that higher frequency of CD4 cell count ≥ 500 cells/mm^3^ (HIV infection, stage 1) (p-value < 0.001) were also significant factors of viral load suppression. The remaining logistic model variables were the following: age (p-value = 0.004), FPL (p-value = 0.031), gender (p-value = 0.328), race/ethnicity (p-value = 0.605), HIV risk factor (p-value = 0.659), and type of medical insurance (p-value = 0.791). We found no significant interactions between housing status and other explanatory variables used in the multivariable binary logistic model. The FPL had a mean of 100.9 and standard deviation of 129.5, while 60.5% of the clients had an annual income under 100% of FPL. As expected, patients living in permanent/stable housing had twice the chance of achieving viral suppression compared with those who were homeless [OR 0.48 (0.32–0.72), p-value < 0.001]. The CD4 count cut-off of 500 cells/mm^3^ was indeed representative of our patient population since 88.2% of them had CD4 count greater than 200 cells/mm^3^. Under the nonparametric assumption, the receiver operating characteristic (ROC) curve plotted in Fig. 1 correctly predicted 72.3% of the primary outcome variable, plasma HIV viral load suppression (AUC = 0.767; p-value < 0.001; 95% CI for AUC (0.73, 0.80). The model is adequate in classifying patients who are virally suppressed and not suppressed.

During the five-year study period, 52 patients died. Of them, 16 (30.76%) were homeless and 36 (69.23%) lived in stable housing, while 12.5% of people experiencing homelessness and 61.12% of those in stable housing achieved viral suppression at the time of death.

## Discussion

Our findings indicate that homelessness, lower retention in care and CD4 cell count less than 500 cells/mm^3^ may be associated with sub-optimal viral suppression. Investigators in San Francisco drew similar conclusions from a surveillance study of 862 HIV-positive residents, showing that homelessness was independently associated with virologic failure (plasma viral load > 200 copies/mL) [5]. A study of 95 homeless PLWHA found an odds ratio of incomplete viral suppression (plasma viral load > 75 copies/mm^3^) 3.84 times higher in people experiencing homelessness compared with housed persons (95% CI 1.36–10.36) [6]. Canadian researchers investigated 922 HIV-seropositive injection drug users and found that longer duration of homelessness was associated with lower odds of viral suppression (adjusted odds ratio = 0.71 per six-month period of homelessness, 95% CI 0.60–0.83) [7]. Researchers in San Francisco discovered a relationship between greater housing instability and lower rates of virologic suppression, ranging from 42% (living outdoors) to 85% (rent/own dwelling) among 1,222 HIV patients [8]*.* The CDC HIV/AIDS Surveillance Project, including 304 homeless across 19 sites throughout the United States demonstrated that PLWHA experiencing homelessness had lower adherence to antiretroviral treatment, lower CD4 cell count, lower likelihood of viral suppression [9]. Another multisite study among 3082 participants in New York, Chicago, Washington, DC, and Los Angeles showed that unstable housing reduced the probability of viral suppression and adequate CD4 cell count by 51% and 53% respectively [10]. Supportive housing program such as Shelter Plus Care, in Cincinnati, Ohio, had achieved statistically significant improvements on CD4 cell count (> 500 cells/mm^3^) and viral suppression (plasma viral load < 200 copies/mL), 45% and 79% respectively [11].

Many studies have shown that incarceration is associated with poor health outcomes among HIV-seropositive adults including greater use of emergency room visits and hospital admissions, and lower prevalence of viral suppression [12, 13]. According to the Brookings Institution, North Nashville has an incarceration rate of 14%, the highest in the country by far, and 93% of those incarcerated are Blacks. In other words, one in seven people who were born in the primary zip code of North Nashville between 1980 and 1986 went to jail or prison at some point in their lives [14]. Twenty five percent of our patients reside in that neighborhood and 41% of them belong to this age group.

Strengths of our study include use of a population disproportionately affected by homelessness and HIV/AIDS, adequate sample size and follow-up duration, and collection of data using validated measures. Nonetheless, this cross-sectional study has several limitations. First, our study has weaknesses inherent to cross-sectional analysis, but we find it reassuring that our results arrive at the consistent message that homelessness contributes to sub-optimal viral suppression in PLWHA. It does not clearly establish homelessness as an independent factor of virologic failure because the study did not address other well-known predictive variables (mental health, substance use disorders, health literacy/numeracy, etc.) in the multivariable binary logistic regression. Nonetheless, a theoretical mathematical model analyzing the effect of homelessness on HIV/AIDS transmission dynamics and comparing housing status-induced reproduction numbers suggests that lack of entertainment, poor nutrition, and co-infection with other sexually transmitted infections in individuals experiencing homelessness may enhance HIV transmission and AIDS-related deaths [15]. The Research on Access to Care in the Homeless (REACH) recruited 104 participants from San Francisco homeless shelters, free-meal programs, and single room-occupancy hotels charging less than $600/month. They discovered that severely food unsecure participants had > 80% lower likelihood of adherence and 77% lower odds of viral suppression (viral load < 50 copies/mL) (95% CI: 0.06–0.82) [16]. A prospective cohort study of 288 HIV-seropositive people experiencing homelessness and unstably housed men, conducted in San Francisco between 2002 and 2008 concluded that the inability to meet food, hygiene, and housing needs was the most powerful factor of physical and mental health, after adjusting for age, race, income, and CD4 cell count [17]. These factors are important because 30% of our patients are facing challenging mental health issues and substance use disorders. More than 50% of our patients did not graduate from high school. The majority of them read at fifth grade level and lack the emotional and familial support to deal with their illnesses in a community with pervasive stigma.

Second, our study does not reflect the racial/ethnic representation of the general population. For example, the proportion of Hispanic/Latinx and transgender communities is relatively small. However, HIV/AIDS and homelessness affect primarily underserved Black/African American communities. Nevertheless, other studies have shown that housing status could be a major factor of HIV health outcomes than demographic features, mental health, substance use disorders, or utilization of other services.

Third, the study does not account for non-infectious comorbidities that could have contributed to higher rates of hospitalizations and emergency room visits, more frequent drug interactions, intolerance, and side effects, leading to sub-optimal treatment adherence and lower rate of viral load suppression, and higher mortality. These critical issues deserve utmost attention because HIV infection may contribute to chronic, sub-clinical inflammation, which promotes the development of cardiovascular and metabolic complications. Notwithstanding the study limitations, multiple researchers have reported the findings that homelessness may be associated with sub-optimal viral suppression.

## Conclusions

Stigmatization and marginalization of individuals experiencing homelessness and HIV-seropositive persons, implicit bias and discrimination, antiquated housing laws, and unfair criminal justice system deserve renewed attention of federal, state, and local authorities. As a public health intervention, housing services align with national medical priorities such as disease prevention and unfettered access to cost-effective and quality health care. At the core of the conceptual framework of the syndemic affecting Black/African Americans in Nashville, Tennessee, HIV/AIDS, homelessness, incarceration, socio-economic status, substance use disorder are intertwined and should be addressed collectively.

## Data Availability

The datasets generated and/or analyzed during the current study are not publicly available due to the personal nature of the data and are not available from the corresponding author upon request.

## Abbreviations

ACA: Affordable Care Act
ACCESS: AIDS care cohort to evaluate exposure to survival services
AIDS: Acquired immunodeficiency syndrome
aRH: Adjusted relative hazard
ART: Antiretroviral treatment
AUC: Area under the curve
CD4: CD4 T lymphocytes
CDC: Centers for disease control and prevention
CI: Confidence interval
FPL: Federal poverty level
HIV: Human immunodeficiency virus
HOPWA: Housing opportunities for people with AIDS
HPTN 052: HIV prevention trials network
IAP: Insurance assistance program
IDU: Injection drug use
iPrEx: Preexposure prophylaxis initiative
IRB: Institutional review board
MAGI: Modified adjusted gross income
MCWC: Meharry Community Wellness Center
MSM: Men having sex with men
OR: Odds ratio
PARTNER: Partners of People on ART—A New Evaluation of the Risks
PCR: Polymerase chain reaction
PEP: Post-exposure HIV prophylaxis
PLWHA: Persons living with HIV/AIDS
PLWH: People living with HIV
PrEP: Pre-exposure HIV prophylaxis
REACH: Research on Access to Care in the Homeless
ROC: Receiver operating characteristic
SARS: Severe acute respiratory syndrome
SARS-COV-2: Severe acute respiratory syndrome coronavirus 2
TGA: Nashville transitional grant area
WIHS: Women’s Interagency HIV Study
